# Estimating the Inbreeding Depression on Cognitive Behavior: A Population Based Study of Child Cohort

**DOI:** 10.1371/journal.pone.0109585

**Published:** 2014-10-14

**Authors:** Mohd Fareed, Mohammad Afzal

**Affiliations:** Human Genetics and Toxicology Laboratory, Section of Genetics, Department of Zoology, Faculty of Life Sciences, Aligarh Muslim University, Aligarh, Uttar Pradesh, India; Oregon Health & Science University, United States of America

## Abstract

**Background:**

Cognitive ability tests are widely assumed to measure maximal intellectual performance and predictive associations between intelligence quotient (IQ) scores and later mental health problems. Very few epidemiologic studies have been done to demonstrate the relationship between familial inbreeding and modest cognitive impairments in children.

**Objective:**

We aimed to estimate the effect of inbreeding on children’s cognitive behavior in comparison with non-inbred children.

**Methodology:**

A cohort of 408 children (6 to 15 years of age) was selected from inbred and non-inbred families of five Muslim populations of Jammu region. The Wechsler Intelligence Scales for Children (WISC) was used to measure the verbal IQ (VIQ), performance IQ (PIQ) and full scale IQ (FSIQ). Family pedigrees were drawn to access the family history and children’s inbred status in terms of coefficient of inbreeding (F).

**Results:**

We found significant decline in child cognitive abilities due to inbreeding and high frequency of mental retardation among offspring from inbred families. The mean differences (95% C.I.) were reported for the VIQ, being −22.00 (−24.82, −19.17), PIQ −26.92 (−29.96, −23.87) and FSIQ −24.47 (−27.35, −21.59) for inbred as compared to non-inbred children (p>0.001). The higher risk of being mentally retarded was found to be more obvious among inbred categories corresponding to the degree of inbreeding and the same accounts least for non-inbred children (p<0.0001). We observed an increase in the difference in mean values for VIQ, PIQ and FSIQ with the increase of inbreeding coefficient and these were found to be statistically significant (p<0.05). The regression analysis showed a fitness decline (depression) for VIQ (R^2^ = 0.436), PIQ (R^2^ = 0.468) and FSIQ (R^2^ = 0.464) with increasing inbreeding coefficients (p<0.01).

**Conclusions:**

Our comprehensive assessment provides the evidence for inbreeding depression on cognitive abilities among children.

## Introduction

The evolution of human brain and cognitive function is driven by different networking or feedback processes underlying genetic and environmental system. Cognitive behavior being an important part of neuroscience that has emerged from quantitative genetic research with intriguing findings and immense implications. Several genetic and epidemiological studies worldwide have documented the risk factors associated with child’s mental health. Cognitive impairments among children, being very much common in developing countries, pose a great challenge in context of medical and public health. Early-onset of cognitive impairment, also known as intellectual disability (ID), commonly referred to as mental retardation (with an IQ below 70), is characterized by significant limitations both in intellectual functioning and the adaptive behavior of the child, which originates before the age of 18 years [1].

A recent study has documented nation-wide cognitive abilities being significantly and substantially correlated with a wide range of phenomena such as educational, economic, political, demographic, sociological, epidemiological, geographic and climatic variables [2]. Although gender based differences in cognitive abilities have been frequently reported and correlated with many factors, there is substantial social and cultural variability in gender equality which is known to be one of the strongest predictor of sex-typed cognitive abilities [3]. Several studies have reported the socio-demographic characteristics such as socioeconomic status (SES), literacy and educational attainment associated with cognitive abilities [4]–[7]. Moreover, quantitative genetic research has revealed surprising findings as most of the environmental measures in social and behavioral sciences show significant and substantial genetic influence [8]–[11].

Quantitative genetic research consistently shows substantial genetic influence on individual cognitive differences in spatial, memory and verbal abilities [12], [13]. The variation in normal human cognition is related to many factors and the sum total of all genetic effects is not likely to be greater than 50% for many types of cognition [14]. Moreover, genes and gene-environment interactions provide substantial pathways for parsing cognitive processes. The two key genetic conceptsS pleiotropy (in which one gene affects many traits) and polygenicity (in which many genes affect a trait) suggest that there is genetic input into the brain structure and function which is general, not modular [15]. With the sequencing of the human genome, it has become possible to identify widespread sequence variations with subsequent heritable risks for common diseases or traits [16]. Approximately, more than 20,000 genes can be considered to play a role in the nurture, plasticity and maintenance of the central nervous system (CNS), although many of these will also play roles in other organ systems as well [17]. The expedition for gene defects underlying cognitive impairment or ID has been much focused on the X-chromosome since the late 19^th^ century, which has ended with molecular elucidation of the fragile X syndrome, the most common form of X-linked ID [18]. Recently, the X-linked recessive gene defects have been revealed to account for about 10 to 12% of the ID seen among the males [19], probably lesser than the erstwhile conjecture [20]. This leaves substantial room for autosomal impact over cognitive impairment, arising either from recessive or dominant gene defects. Still, compared with X-linked ID (XLID), the molecular elucidation of the autosomal recessive ID (ARID) has lagged far behind, which provide ample scope for rather different homozygosity studies.

Inbreeding (consanguineous marriages among humans) produces homozygous offspring, since the mating of pairs occurs between genetically closely related individuals. The phenomenon of inbreeding or endogamy, increases the level of homozygotes for autosomal recessive genetic disorders and generally leads to decreased fitness of a population known as inbreeding depression which provides a major focus in clinical studies [21]. The inbreeding depression that results from such consanguineous mating is mostly a consequence of additive effect of recessive alleles in the homozygous condition [22]. Parental consanguinity has been associated with increased risk of adverse prenatal outcomes including stillbirths, low birth weight, preterm delivery, abortion, infant and child mortality, congenital birth defects, cognitive impairments, malformations and many other complex disorders [12]–[32]. A study has revealed that the overall incidence of congenital malformations was 2.5 times higher amongst the children of inbred families when compared to that of non-inbred families [33]. Consanguinity has been associated with significant decline in mean values for height, weight and body mass index (BMI) and the subsequent depression on children growth, much influenced in proportion to their inbreeding coefficients with least variation for non-genetic factors [21]. Inbreeding is also thought to predispose offspring to neuropsychological disorders such as hereditary Parkinsonism [34]. Several studies have identified parental consanguinity as an important risk factor for mental retardation or ID [26], [27], [35], and it has been shown that inbreeding is correlated with reduced cognitive performance [36].

The determination of major medical conditions in early childhood due to consanguinity is an important component of clinical genetic studies. The populations of India and other South Asian countries, which have many Muslim communities, offer great opportunities for studying the influence of socio-cultural and genetic variability on consanguinity and child health. North Indian populations and those from Jammu and Kashmir (J&K) have historical, linguistic, cultural, and socio-religious significance for the Indian subcontinent [37]. The incidence of consanguineous marriages ranges from 35% to 50% among the populations under study. One of our recent studies taken among the same populations showed higher frequency of homozygosity for Rhesus protein genes than that found elsewhere in the Indian subcontinent [38]. The present study, done for the first time in Jammu region, can amount to making a preliminary effort toward realization of genetic hazards due to inbreeding. Such a study is of great help for genetic counseling and the basics of genetic epidemiology in the region. The aim of the present study was to explore the effect of inbreeding among children from northernmost part of Indian subcontinent for cognitive abilities (IQ) in comparison with non-genetic factors. Analysis of pedigree was done to estimate the coefficient of inbreeding (F), followed by the observation of cognitive impairment trends among children from Muslim populations of inbred and non-inbred families.

## Methods

### Ethics Statement

The study was approved by the Institutional Ethics Committee of Jawaharlal Nehru Medical College (JNMC), Aligarh Muslim University, India. We obtained informed written consent from the parents, caretakers, or guardians on behalf of minors/children participants involved in our study.

### Population and Study design

The Muslim populations were selected in our survey from Rajouri and Poonch Districts of J&K for two reasons: firstly, consanguineous marriages are favored among Muslim families, while the Hindu and Sikh religions avoid marriages among close relatives; secondly nearly 70% of the total population is Muslim in the region surveyed. In order to select the subjects for their IQ studies, preliminary visits to the areas were made and it was found that more than 75% (approx.) population lives in villages, which is mainly due to topographical feature of the area (hilly region), which thus makes it less urbanized. We also observed the culture and beliefs related with marriages among different populations. The castes and tribes were very rigid, favoring marriages among close relatives or those within the caste, which in turn also increases the rate of inbreeding.

**Figure 1:**
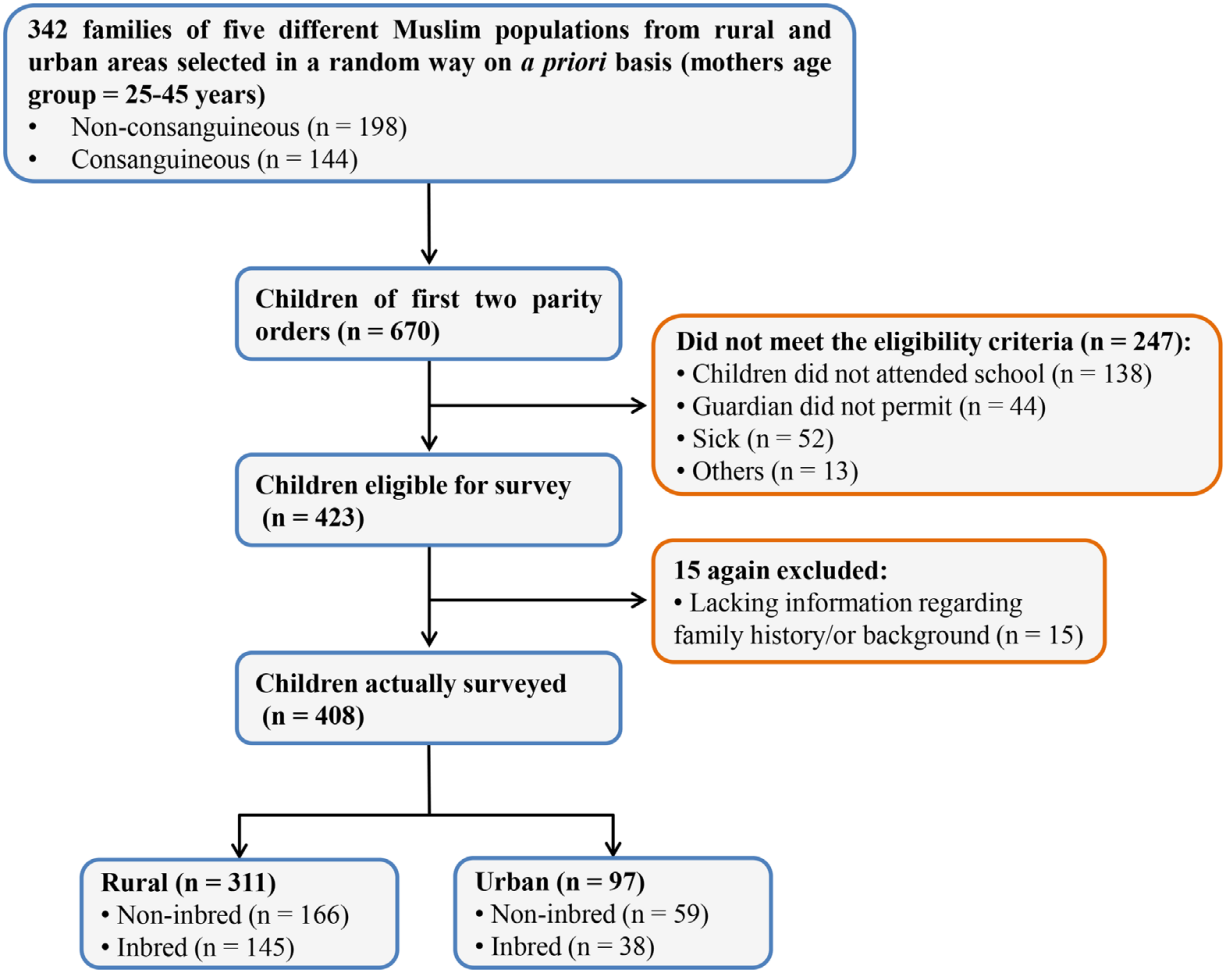
Study design. Flowchart depicting the steps involved in the recruitment process.

The study was conducted during April 2013 through July 2013 and a total of 408 children (6 to 15 years of age) were selected randomly on *a priori* basis from five Muslim populations viz., Gujjar and Bakarwal (n = 97), Mughal (n = 72), Malik (n = 86), Syed (n = 65) and Khan (n = 88). The age group of the children selected was 6 to 15 years. Children from non-consanguineous marriages (either belonging to the same family or from other families of the same population) served as control. Only the first two parity-order children were taken. Those having prolonged illness or suffering from congenital defects were excluded. Children with no schooling were also excluded. Figure 1 depicts the steps involved in the recruitment process. The details of sample size and variables under study are presented in Table 1.

**Table 1:** Details of sample size and selection of categories in the study.

Factors	Categories	Non-inbred	Inbred	Total (n)
**Residence**	Rural	166 (53.38%)	145 (46.62%)	311
	Urban	59 (60.82%)	38 (39.18%)	97
**SES**	High	52 (61.90%)	32 (38.10%)	84
	Medium	111 (54.15%)	94 (45.85%)	205
	Low	62 (52.11%)	57 (47.89%)	119
**Population**	Gujjar and Bakarwal	55 (56.70%)	42 (43.30%)	97
	Mughal	41 (56.94%)	31 (43.06%)	72
	Malik	47 (54.65%)	39 (45.35%)	86
	Syed	35 (53.85%)	30 (46.15%)	65
	Khan	47 (53.41%)	41 (46.59%)	88
**Age (in years)**	6	27 (56.25%)	21 (43.75%)	48
	7	21 (55.26%)	17 (44.74%)	38
	8	35 (57.38%)	26 (42.62%)	61
	9	10 (55.56%)	08 (44.44%)	18
	10	35 (58.33%)	25 (41.67%)	60
	11	14 (56.00%)	11 (44.00%)	25
	12	24 (46.15%)	28 (53.85%)	52
	13	11 (50.00%)	11 (50.00%)	22
	14	22 (68.75%)	10 (31.25%)	32
	15	26 (50.00%)	26 (50.00%)	52
**Gender**	Male	105 (55.85%)	83 (44.15%)	188
	Female	120 (54.55%)	100 (45.45%)	220
	Combined	225 (55.15%)	183 (44.85%)	408

Values presented as number and percentage (in parentheses) against different categories.

Total sample size 408, n = number of subjects.

### Parental background

The parental background characteristics were assessed in two dimensions: the residence (rural or urban) and the socioeconomic status (SES; low, medium and high). The indigenous adapted Kuppuswamy’s socioeconomic status scale [39] was used in this study (Table 2). The parental SES was based on three domains: education, occupation and household income. SES was assessed for both the parents (i.e., mother and father) and mean scores on the whole were taken for complete parental SES. On the basis of scores, we categorised SES into three classes: 26–29 as high, 11–25 as medium and <10 as low. All the information was recorded in a standardised questionnaire.

**Table 2:** Characteristics of socioeconomic status (SES) scale used in the study.

Education	Score	Occupation	Score	Income	Score
Profession or honours	07	Profession	10	≥30375	12
Graduate or post graduate	06	Semi-profession	06	15188−30374	10
Intermediate or post high school	05	Clerical, shop-owner, farmer	05	11362−15187	06
High school certificate	04	Skilled worker	04	7594−11361	04
Middle school certificate	03	Semi-skilled worker	03	4556−7593	03
Primary school certificate	02	Unskilled worker	02	1521−4555	02
Illiterate	01	Unemployed	01	≤1520	01

### Pedigree analysis

Genealogical information (family pedigrees) up to five generations back (volunteered by the parents) helped in ascertaining the consanguinity persuasion of their marriage and thus, child’s inbreeding status was determined. The information provided by the parents was cross checked by seeking help from the elder members of the family. In case of ambiguity such data were omitted.

Wright’s path relationship method for calculating the coefficient of inbreeding (F) was used for all mating types following the order: double first cousin (F = 0.125) > first cousin (F = 0.0625) > first cousin once removed (F = 0.03125) > second cousin (F = 0.0156). In non-consanguineous families or for the distant marriages, the coefficient of inbreeding was effectively zero (F = 0.000) [21].

### Measures

The Wechsler’s Intelligence Scales exists in two forms, Wechsler’s Adult Intelligence Scale (WAIS) and Wechsler’s Intelligence Scales for Children (WISC). For determining the IQ of children below 16 years, the WISC test system is used. We used Indian adapted WISC-III (3^rd^ edition), which is made up of a battery of ten subtests consisting of five verbal and five performance tests as follows:

### Verbal IQ tests

- *Information:* General knowledge based questions.
- *Comprehension:* Questions based on social situations or common concepts.
- *Arithmetic:* Orally administered arithmetic questions.
- *Vocabulary:* Examinee was asked to define a provided word.
- *Digit span:* Children were orally given sequences of numbers and asked to repeat them, either as heard or in reverse order.

### Performance IQ tests

- *Picture completion:* Children were shown artwork of common objects with a missing part, and asked to identify the missing part by pointing and/or naming.
- *Block design:* Children have to put together red and white blocks in a pattern according to the model displayed. This was timed, and some of the more difficult puzzles result into the award of bonus scores for speed.
- *Object assembly:* Children were provided with pieces of four different objects such as man, horse, auto and human face. For perfect performance, full scores as well as bonus is given. Imperfect performance like inverting or omitting pieces leads to negative marking.
- *Mazes:* Children were shown the samples and with the help of pencil a demonstration was given. The test was discontinued if two consecutive failures were found.
- *Coding:* Children under 8 years of age could mark rows of shapes with different lines according to a code, whereas children over eight transcribe a digit-symbol code. The task was time-limited, again with a bonus for speed.

Each of the above subtests consists of many items, each item carries a quantum of raw score (1, 2, 3, and so on) upon completion within a given fraction of time (in seconds). The sum total of these scores gives a raw subset score, which was then converted into IQ score by using WISC manual. The means of the subsets of the two categories (verbal and performance), gives VIQ and PIQ and the overall mean (of VIQ and PIQ) gives FSIQ. Table 3 presents the classification of the subjects on the basis of their FSIQs on WISC-III manual. The medium of the test was English as no Urdu or Hindi versions were available. So the verbal tests were translated into Urdu by the author, with slight alterations in their cultural reference wherever necessary. Such alterations were kept to a minimum (two occasions), one each in information and comprehension. Alterations in the performance subsets were not needed.

**Table 3:** Classification of intelligence based on WISC-III scores.

IQ score	Classification
130 and above	Very Superior
120−129	Superior
110−119	High Average
90−109	Average
80−89	Low Average
70−79	Borderline
55−69	Mild Mental Retardation
40−54	Moderate Mental Retardation

### Procedure

The subjects were approached through their parents and their name, age, sex, class and school attended with parental consanguinity were entered in the record form. The children were asked questions from the questionnaire as per instructions given in the manual and their answers were then recorded on the schedule. Each child performed the test individually in a quiet room at their home, which lasted for about one hour depending upon the intelligence and/or cooperation from the candidate. The VIQ and PIQ subtests were performed in a sequence as per guidelines. Timing was noted with a stop-watch. While giving the tests, full rapport was established with the child and every effort was made to engage his attention. Unnecessary interference by parents or by brothers/sisters and others in the household was avoided. Some encouraging words were also used and appreciating gestures were made to keep the enthusiasm of the child. Those who ran away after sitting for a while were approached later, while those who began to cry or kept mum were omitted. The scoring was done immediately after the test. The raw scores thus collected, were later converted into scaled scores with the help of the WISC-III manual.

### Statistical analysis

Statistical analysis was conducted using SPSS software 17.0 version (SPSS, Chicago, IL, USA) and GraphPad InStat 3.0 (USA). Data are presented as mean±SD and mean±SE for comparing the results using different variables (residence, SES, age, gender and inbreeding). For FSIQ categories (scores range: ≥130 to 40−54), chi-square (χ^2^) test was used to determine the significant differences. The differences in mean values among inbred categories from the non-inbred (as controlled) children for VIQ, PIQ and FSIQ (95% Confidence Interval) were analyzed using post-hoc tests (Bonferroni and Tukey HSD) and p-value <0.05 was considered for the level of significance. ANOVA-test for four factor analysis on VIQ, PIQ and FSIQ was used and p-values were compared for significant results. Regression analysis was done to estimate inbreeding depression on VIQ, PIQ and FSIQ and ‘R^2^’ values were observed at p<0.01. The inbreeding depression on cognitive abilities (VIQ, PIQ and FSIQ) was calculated using average coefficient of inbreeding (*α*) and percentage of depression in trait for ‘*α*’ [21], [27], [40] as follows:
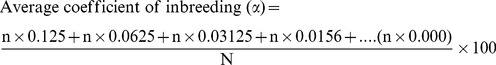
(1)

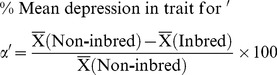
(2)

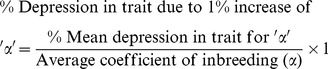
(3)


In the first equation, ‘n’ indicates number of individuals multiplied with inbreeding coefficients (F) for each of the inbreeding category. The F-value = 0.000 indicates simply non-inbred individuals while N is sum total of the individuals. The 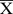 in the second indicates the mean value of the trait.

## Results

### Descriptive characteristics of cognitive abilities with inbreeding

**Figure 2:**
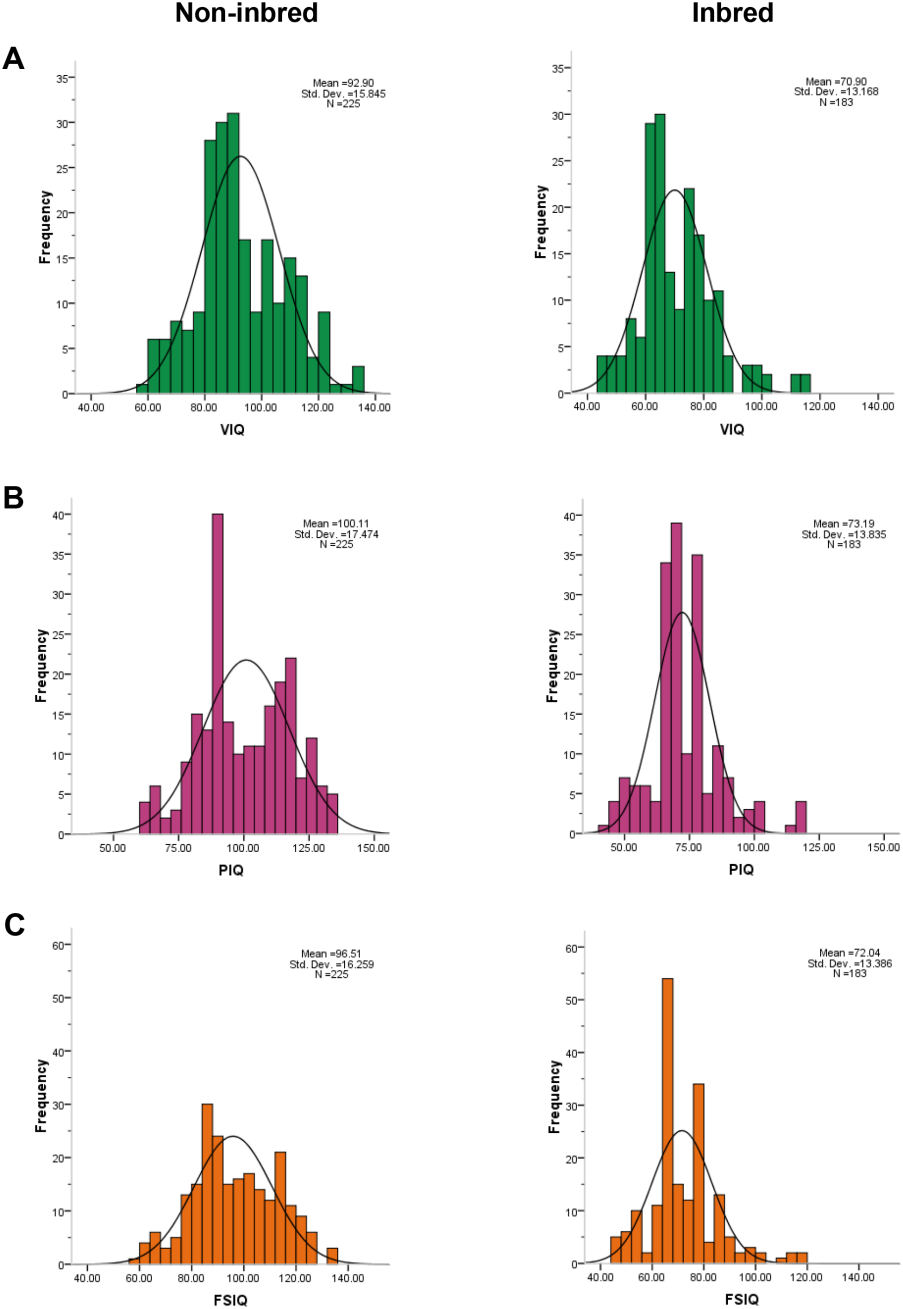
Normal distribution of cognitive abilities among inbred in comparison with non-inbred children. Differences in normal distributions of inbred in comparison with non-inbred children for VIQ, PIQ and FSIQ show highly significant results (at p<0.0001, using One-way Analysis of Variance). The mean difference (95% CI) for VIQ −22.00 (−24.82, −19.17), PIQ −26.92 (−29.96, −23.87) and FSIQ −24.47 (−27.35, −21.59) among inbred groups in comparison with non-inbred found to be statistically significant (at p<0.001, using Tukey-Kramer Comparisons Test). The non-inbred shows a regular pattern, whereas frequency distributions among inbred children represent a shift toward lower values and sparsity toward the high average or superior IQs.

Figure 2 presents an overview of the normal distribution for cognitive abilities (VIQ, PIQ and FSIQ) among inbred and non-inbred children. The frequency polygon for inbred children showed a shift toward low cognitive abilities as compared to non-inbred group (i.e., increased frequency of mental illness among inbred as compared to non-inbred children). We found a significant difference for VIQ, PIQ and FSIQ between inbred and non-inbred children (at p<0.0001, using ANOVA). The mean difference (95% CI) in terms of VIQ −22.00 (−24.82, −19.17), PIQ −26.92 (−29.96, −23.87) and FSIQ −24.47 (−27.35, −21.59) for inbred children compared with non-inbred children were found to be statistically significant (p<0.001, using Tukey-Kramer Comparisons Test).

**Figure 3:**
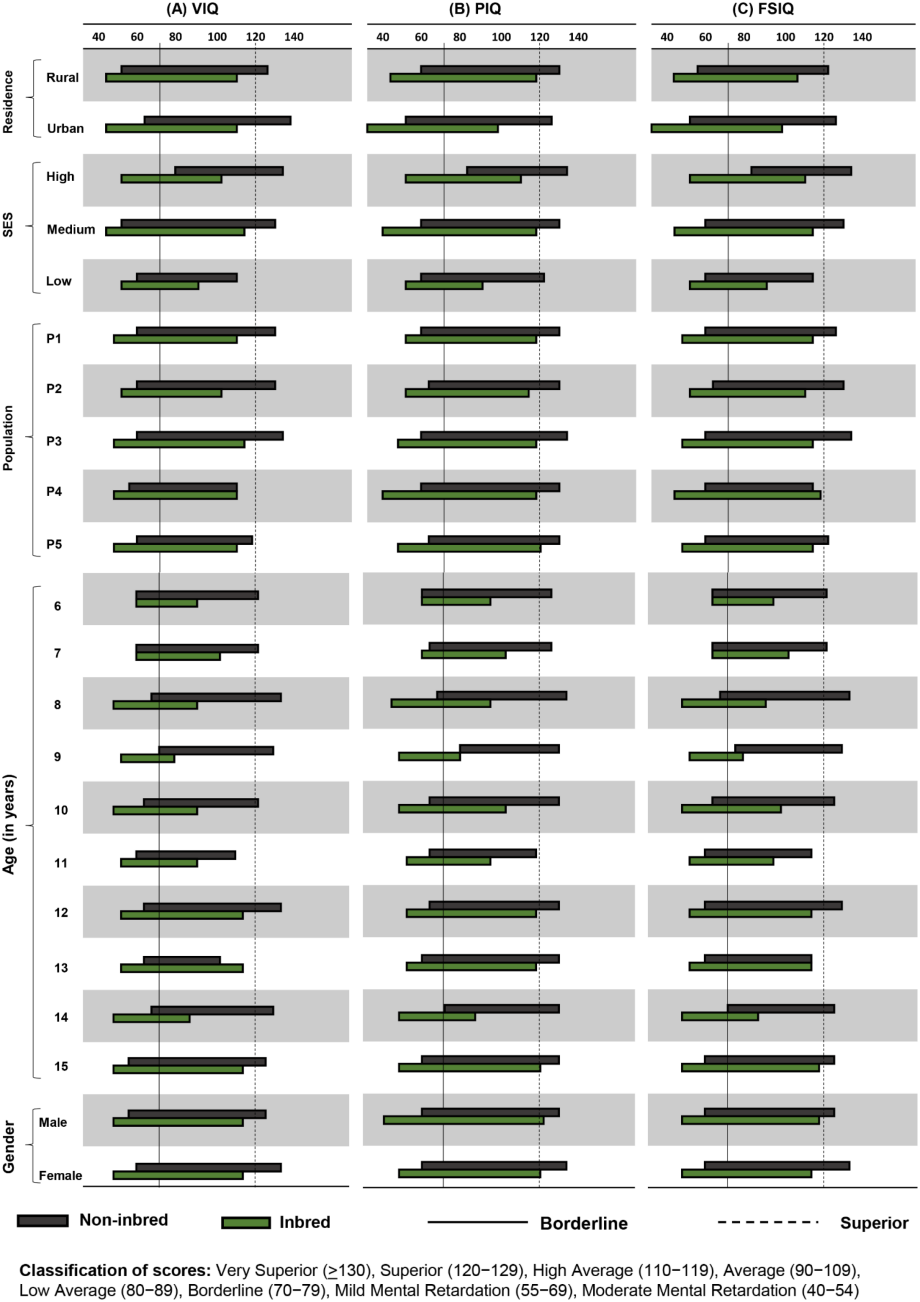
Range distribution of cognitive abilities for different factors. The IQ range has been established with minimum and maximum values observed for each category. Among all factors, inbred subjects exhibit a range shift toward low IQ, not crossing the superior IQ which is the indication of inbreeding load on the child’s cognitive abilities to have elevated levels of mental illness. Factors, like residence, SES and population (except age and sex) show significant differences though overall effect yet remained incompetent in comparison with inbreeding. The populations presented as, P1 = Gujjar and Bakarwal, P2 = Mughal, P3 = Malik, P4 = Syed, P5 = Khan.

Figure 3 shows the range and distribution of IQ, assessing the cognitive ability differences among inbred and non-inbred children for different factors. The inbred subjects lie largely toward average to borderline IQ scores, not passing the ambit of superior IQs in case of all factors. The overall picture showed inbred subjects exhibit a cognitive shift toward low IQ, while non-inbred group presented the balanced distribution with a turn toward high cognitive abilities.

### Cognitive differences among inbred and non-inbred children

**Figure 4:**
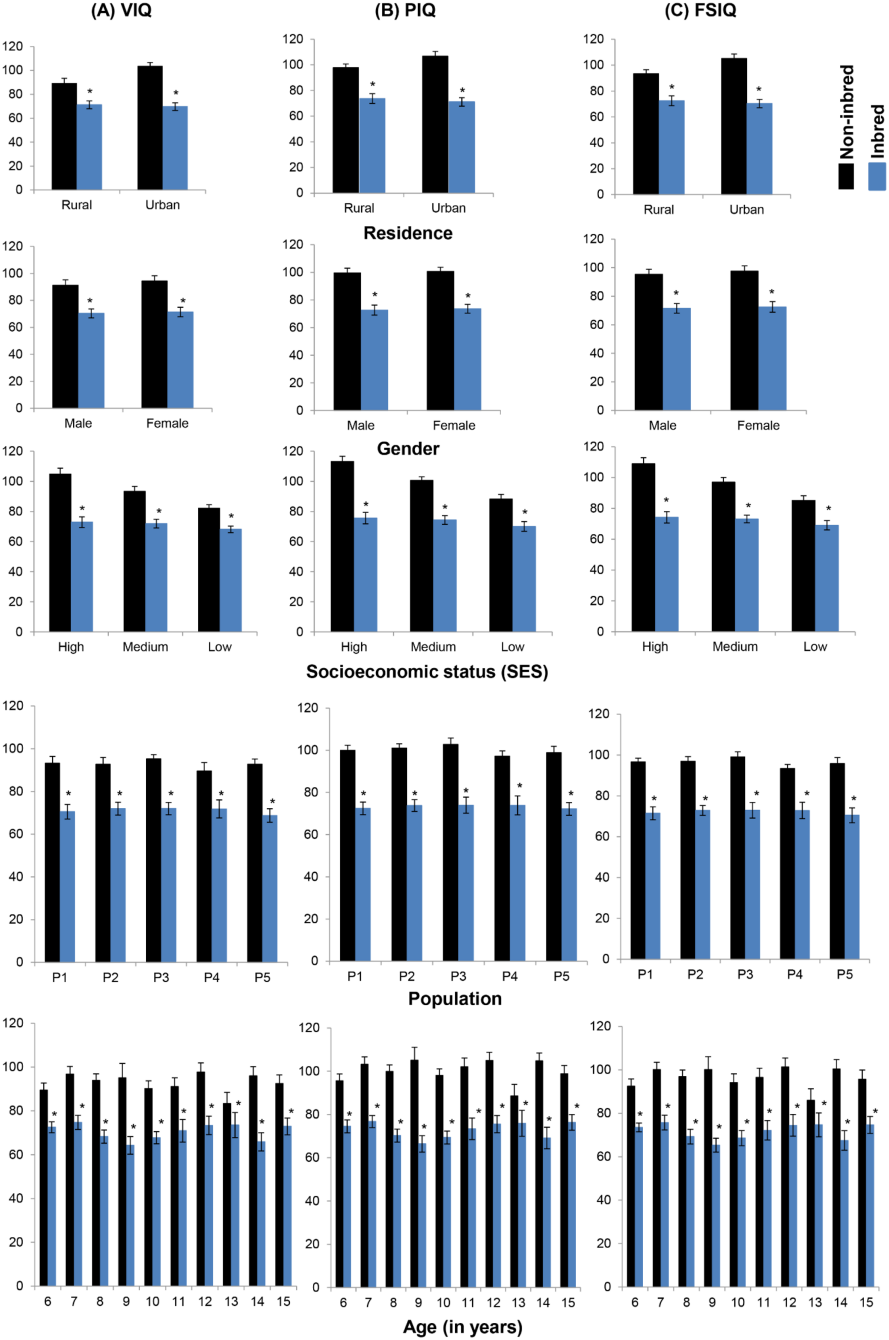
Cognitive abilities among inbred and non-inbred children. The results presented as mean±SEM. The significant decline in mean values observed for VIQ, PIQ and FSIQ among inbred children in comparison with non-inbred group. *The difference in mean values of inbred from non-inbred children for each factor is found to be statistically significant (p<0.05, using student’s t-test). Populations presented as, P1 = Gujjar and Bakarwal, P2 = Mughal, P3 = Malik, P4 = Syed, P5 = Khan.

Figure 4 presents the differences in cognitive abilities among inbred and non-inbred children across different factors. The inbred children showed significant decline in mean values as compared to non-inbred group for all the factors (p<0.001). Despite the fact, non-inbred subjects showed slight differences in their intelligence for residence (urban > rural) and SES (high > medium > low), yet their effect remained non-significant among inbred subjects, which indicate the cognitive ability being strongly suppressed as a result of inbreeding. Factors other than inbreeding, like population, age and sex showed inconsequential differences. This provides the indication of inbreeding depression (a genetic component) having much influence over cognitive parameters than other factors.

### Factor analysis for cognitive abilities

Table 4 presents summary statistics based on ANOVA test for factor analysis. To investigate the strong cognitive predictor, we hypothesized two models (Model I and Model II). In both the models, age and inbreeding were taken as common and constitute a total of four factors, to predict the level of significance for each factor. In model I, inbreeding was found significant whereas population, sex and age presented non-significant association with VIQ, PIQ and FSIQ. In model II, again only inbreeding was found as a prominent factor significantly associated with all cognitive tests whereas residence, SES and age were remained insignificant. These two models strongly support the cogent impact of inbreeding on cognitive abilities of children.

**Table 4:** ANOVA results of four factor analysis based on mean values of verbal, performance and full scale IQ.

	Model I						Model II					
	Source	df	ss	ms	F-value	p-value	Source	df	ss	ms	F-value	p-value
**VIQ**												
	Population	04	546.08	136.52	0.0645	0.9917	Residence	01	1084.5	1084.5	0.9987	0.3316
	Gender	01	4.43	4.43	0.0021	0.7017	SES	02	3268.973	1634.4865	1.5052	0.2501
	Age	09	13325.65	1480.62	0.6998	0.9639	Age	09	8002.68	889.18	0.8188	0.6072
	Inbreeding	01	27954.30	27954.30	13.2126	0.0018***	Inbreeding	01	14721.29	14721.29	13.5575	0.0018***
	Within Groups	19	40198.71	2115.72			Within Groups	17	18459.34	1085.84		
**PIQ**												
	Population	04	350.81	87.70	0.02926	0.9982	Residence	01	191.9005	191.9005	0.1264	0.7266
	Gender	01	6.11	6.11	0.00203	0.8428	SES	02	4424.3895	2212.1947	1.4573	0.2605
	Age	09	14002.61	1555.84	0.51911	0.9645	Age	09	8671.4005	963.4889	0.6347	0.753
	Inbreeding	01	39858.22	39858.22	13.2988	0.0017***	Inbreeding	01	21173.8995	21173.8995	13.9491	0.0016***
	Within Groups	19	56945.08	2997.11			Within Groups	17	25804.901	1517.9353		
**FSIQ**												
	Population	04	464.86	116.21	0.04619	0.9956	Residence	01	546.69	546.69	0.42709	0.5222
	Gender	01	1.59	1.59	0.00063	0.8167	SES	02	3828.1561	1914.07805	1.4953	0.2522
	Age	09	12555.44	1395.05	0.5545	0.9802	Age	09	8091.6296	899.0699	0.70239	0.6991
	Inbreeding	01	33628.87	33628.87	13.3668	0.0017***	Inbreeding	01	17808.3793	17808.3793	13.9127	0.0017***
	Within Groups	19	47801.26	2515.85			Within Groups	17	21760.1104	1280.0065		

Model I = Population×Gender×Age×Inbreeding, Model II = Residence×SES×Age×Inbreeding.

df = degrees of freedom; ss = sum of squares; ms = means square; SES = socioeconomic status.

In Model I, the population, gender, and age based differences are found to be non-significant for all cognitive tests.

In Model II, the residence, SES and age are found to be insignificant.

Inbreeding is found to be significant for all cognitive parameters in both the models.

***Statistically significant at p<0.001.

### Inbreeding coefficient step-up and cognitive abilities

Table 5 presents the mean difference in cognitive abilities among inbred children as compared to control group (i.e., non-inbred children). With the increase of inbreeding coefficient (i.e., the inbred categories; F = 0.0156 to 0.125), there was found a persuasive decline in mean values for cognitive parameters in comparison with non-inbred group (F = 0.00). The mean difference in VIQ, PIQ and FSIQ showed a significant inflation with the increase of the degree of inbreeding (p<0.05, using post-hoc tests) and followed the order: double first cousin > first cousin > first cousin once removed > second cousin > non-inbred. This indicates the risk for cognitive impairments or ID was more common among the subjects having greater value of inbreeding coefficients.

**Table 5:** Mean differences in cognitive abilities among inbred categories in comparison with non-inbred.

Dependent Variable	F	Mean±SD	Mean Difference±SE (95% CI)
**VIQ**	0.00	92.89±15.84	
	0.0156	86.77±13.92	−6.12±2.58 (−0.97, 13.21)
	0.03125	78.87±6.20	−14.02±3.08* (5.56, 22.46)
	0.0625	67.66±7.57	−25.23±1.59* (20.85, 29.61)
	0.125	58.62±13.90	−34.27±2.79* (26.60, 41.94)
**PIQ**	0.00	100.11±17.47	
	0.0156	90.38±14.18	−9.72±2.82* (1.99, 17.46)
	0.03125	80.53±7.16	−19.57±3.36* (10.36, 28.78)
	0.0625	69.79±7.18	−30.31±1.74* (25.53, 35.08)
	0.125	60.43±16.34	−39.67±3.05* (31.31, 48.04)
**FSIQ**	0.00	96.51±16.25	
	0.0156	88.57±13.99	−7.93±2.64* (0.68, 15.18)
	0.03125	79.70±6.51	−16.80±3.15* (8.16, 25.44)
	0.0625	68.72±7.15	−27.78±1.63* (23.31, 32.25)
	0.125	59.52±14.99	−36.98±2.86* (29.14, 44.82)

Results for mean differences in verbal, performance and full scale IQ of inbred groups (F = 0.0156 to 0.125) from non-inbred (F = 0.000) obtained by using post-hoc tests (Bonferroni and Tukey HSD).

The values for mean difference show significant increase (indicating inbreeding depression) with the increase of the coefficient of inbreeding (F).

Negative sign represents the decrease in mean difference from control one (i.e., non-inbred).

F = Coefficient of inbreeding, CI = Confidence Interval (Lower bound, Upper bound), SE = standard error.

*Significantly different from control (i.e., non-inbred) at p<0.05.

**Figure 5:**
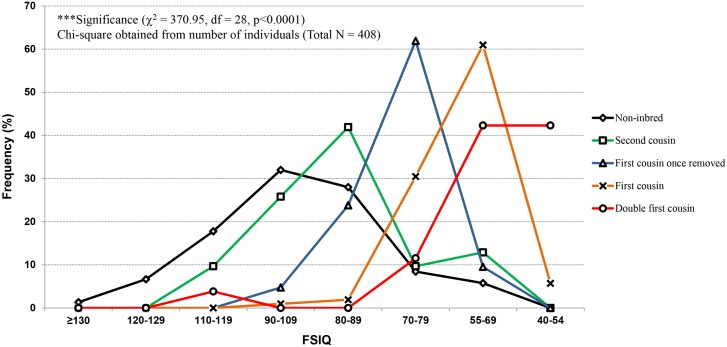
FSIQ comparisons in respect to the degree of inbreeding. The results depict the percentage frequency distribution of FSIQ in relation to coefficient of inbreeding. The non-inbred children display high frequency peaks on left side (presenting high FSIQ values) and downturn toward low FSIQ scores. On the other hand, elevated peaks are observed for low FSIQ scores (on right side) with the increase of inbreeding coefficient (F = 0.0156 to 0.125) and flattened toward high FSIQ scores, providing the evidence for inbreeding depression on children FSIQ.

The frequency distribution (in percentage) of children based on FSIQ scores for each category was examined (Figure 5). The line graph presents the significant difference (p<0.0001) in frequency distribution of FSIQs among inbred categories correlated with non-inbred group. For mental retardation (FSIQ = 55–69 or 40–54), the lowest frequency was observed in case of non-inbred children, whereas inbred children with higher inbreeding coefficients presented higher frequency of mental retardation. The line graph for inbred subjects gets flattened toward high FSIQ scores (i.e., high average, superior, and very superior), whereas it showed the high frequency peaks toward lower FSIQ scores (i.e., borderline and mental retardation).

### Inbreeding depression on cognitive behavior

**Figure 6:**
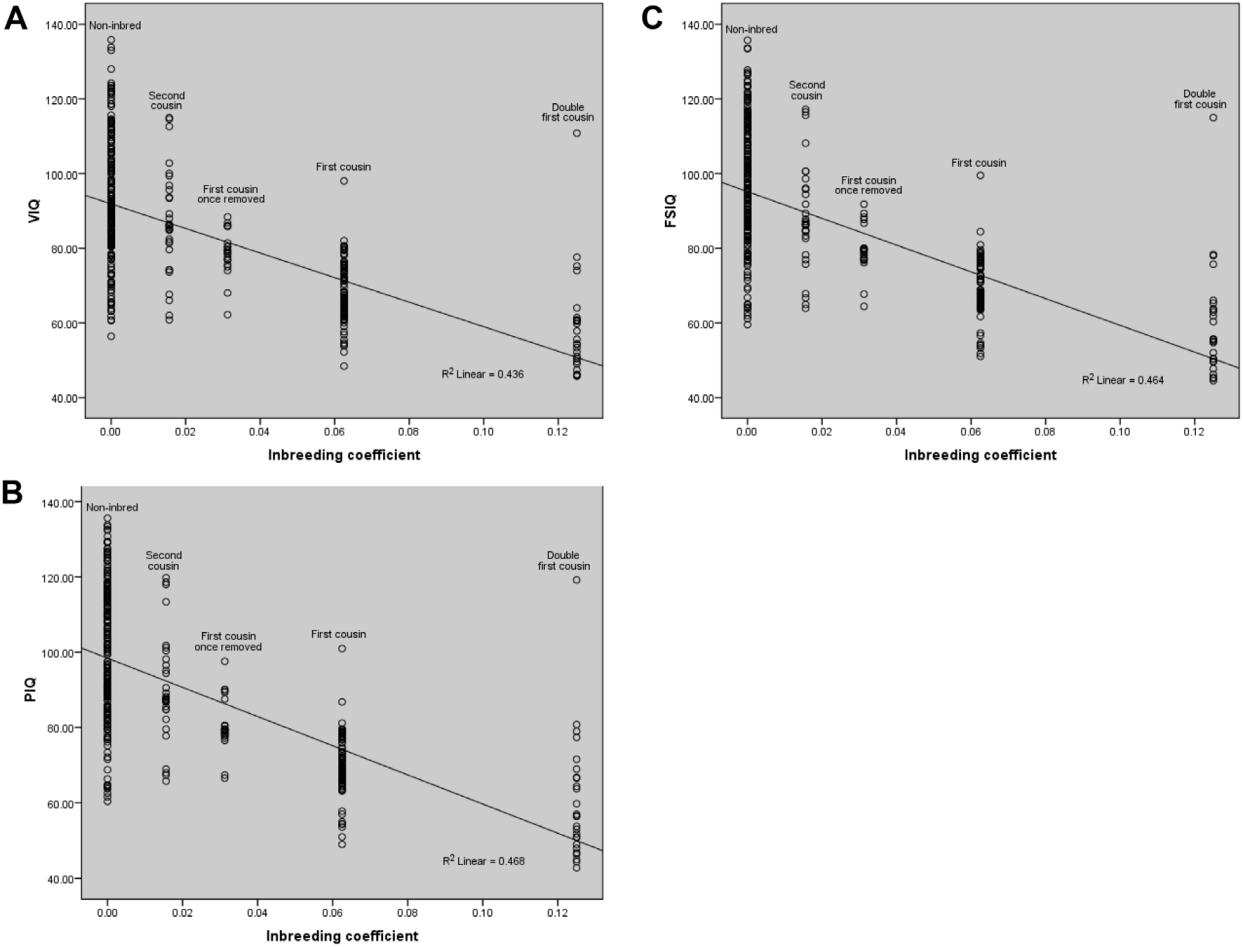
Inbreeding depression on cognitive abilities. The regression analysis presents almost similar trend for VIQ, PIQ and FSIQ. The slope depicts fitness decline (regression slope) with increase of inbreeding coefficient (F). The observed linear decline in fitness for cognitive parameters; (A) VIQ (R^2^ = 0.436), (B) PIQ (R^2^ = 0.468) and (C) FSIQ (R^2^ = 0.464) are found to be significant. Pearson correlation ‘r’ for VIQ, PIQ and FSIQ are −0.660, −0.684 and −0.681 respectively, and found to be statistically significant (p<0.01). The negative values indicate the decreasing trend for cognitive abilities due to inbreeding.

Besides the differences in percentage and mean values for VIQ, PIQ and FSIQ for different factors, we found relatively similar trend in regression model with increasing inbreeding coefficients (Figure 6). The regression slope shows a fitness decline with the increase of inbreeding coefficient. The observed linear decline in fitness for VIQ (R^2^ = 0.436), PIQ (R^2^ = 0.468) and FSIQ (R^2^ = 0.464) were found to be statistically significant (p<0.01). Moreover, regression analysis showed Pearson correlation ‘r’ for VIQ, PIQ and FSIQ as −0.660, −0.684 and −0.681, respectively, all of which were statistically significant at p<0.01. The negative sign for r-value indicates the depression in cognitive tests with the increasing levels of inbreeding. The regression analysis strongly supports the evidence of inbreeding depression on child’s verbal, performance and full intellectual skills.

Table 6 presents the summary statistics for average coefficient of inbreeding and percentage of inbreeding depression on cognitive parameters. The average coefficient of inbreeding was almost the same for all of the factors except SES. The average coefficient of inbreeding among the subjects followed the order: low > medium > high SES families. This suggests that children from higher SES tend to have low inbreeding (*a* = 1.93), which is due to parental education and awareness for marriage choice, being not much confined to social norms, whereas lower SES depicts the children from higher inbreeding level (*a* = 3.06), suggesting a lower awareness or lack of education as well as adherence to social beliefs among parents for marrying within families/castes. The percentage depression for all IQ values among children in case of high inbreeding coefficients provide greater risk of being mentally retarded (or ID).

**Table 6:** Average coefficient of inbreeding and percentage inbreeding depression on cognitive abilities.

Factors	Categories	Averagecoefficient ofinbreeding (*α*)	% Depressionin VIQ for *α*	% Depressionin VIQ due to1% increase ofinbreedingcoefficient	% Depressionin PIQ for *α*	% Depression inPIQ due to 1%increase of inbreedingcoefficient	% Depressionin FSIQ for *α*	% Depression inFSIQ due to 1%increase ofinbreeding coefficient
**Residence**	Rural	2.78	20.12	7.23	24.56	8.83	22.45	8.07
	Urban	2.17	32.63	15.03	33.37	15.37	33.01	15.21
**SES**	High	1.93	30.41	15.75	33.23	17.21	31.86	16.51
	Medium	2.69	22.96	8.53	26.14	9.71	24.62	9.15
	Low	3.06	17.11	5.59	20.67	6.75	18.94	6.19
**Population**	Gujjar andBakarwal	2.53	24.34	9.62	27.52	10.87	24.56	9.71
	Mughal	2.32	22.41	9.65	26.99	11.63	24.79	10.68
	Malik	2.61	24.43	9.36	28.04	10.76	26.34	10.09
	Syed	2.71	19.78	7.29	24.02	8.86	21.99	8.11
	Khan	3.01	25.86	8.59	26.94	8.95	26.42	8.77
**Age (in** **years)**	Combined(6−15)	2.64	23.67	8.96	26.89	10.18	25.35	9.61
**Sex**	Male	2.71	22.81	8.41	27.01	9.96	25.01	9.22
	Female	2.57	24.42	9.51	26.81	10.43	25.68	9.99

Almost all the factors shows the same trend of inbreeding depression viz. PIQ > FSIQ > VIQ, though there is a significant difference among the categories of each factor.

## Discussion

Parental consanguinity leads to inbreeding which often result into genetic drift, and hence may swiftly give rise to evolutionary consequence to pose the most immediate risk [41]. Inbreeding exposes the action of deleterious recessive alleles and reduces fitness in terms of loss of heterozygosity [22]. The increased homozygosity (due to inbreeding) implies lower genetic variation that may lead to the expression of recessive alleles, or loss of hybrid vigor, of essential genes for the traits and thus may lead to the cause of depression (fitness decline) among inbred populations [21]. The harmful effects of inbreeding were first documented and quantified in detail by Charles Darwin, who carried out experiments on 57 plant species that involved self-fertilization (inbreeding) and outcrossing between unrelated groups. Darwin’s experiments supported his hypothesis that self-fertilization must be strongly disadvantageous for the progeny produced– it lowered vigor and fertility in most of his species studied [42]. Further, inbreeding experiments carried out in maize plant has exposed the cause of depression in growth and vigor among selfed generations [43]. Increased frequency of polydactyly has also been reported among inbred strains of guinea pigs [44]. The majority of studies done on human beings support the relationship between inbreeding and the increased risk for recessive and many other complex traits. The pioneers of human inbreeding, Schull and Neel [45], [46] while studying children of atomic bomb survivors in Japan after the World War II, found higher mortality, increased rate of genetic disorders, and many other health related hazards, more often among inbred children in comparison with the non-inbred group. The relative abundance of recessive disorders among inbred communities of many Arab countries has been associated clearly with the practice of consanguinity [23], [47].

In the present study, coefficient of inbreeding (a measure of homozygosity) has been calculated through standard pedigree method. Higher value of inbreeding coefficients presents more homozygosity and respective cognitive behaviors have been studied. Based on the same hypothesis, a number of recommendations (such as large population size, different degrees of inbreeding against control groups and well-developed statistical methodology) have been suggested for the optimal design of experiments to study genetic drift and inbreeding as well as correction of data for general environmental effects [48]. A recent study has provided an evidence of inbreeding depression on growth parameters of children with higher frequencies of underweight (BMI <18.5 kg/m^2^) cases with increasing inbreeding coefficients [21]. The regression based fitness-related model has been used to quantify the inbreeding depression on cognitive behavior. Similarly, inbreeding load with increased inbreeding coefficients has also been made clear in earlier studies [21], [22], [49], [50].

This study is one of the first attempts with details of inbreeding coefficients and elaborate statistics to examine the effects of inbreeding on the cognitive abilities of children among the indigenous Muslim populations of Jammu region (Northern India). Our results showed a significant decline in cognitive abilities of children due to inbreeding and higher frequency of mental retardation observed among offspring of inbred families, whereas children from non-consanguineous families display higher values of VIQ, PIQ and FSIQ scores and consequently low frequency of mental retardation or ID. Various studies on consanguineous marriages and other forms of inbreeding have cited a discernible reduction in cognitive abilities, with increased mental illness in the offspring of such unions [26], [51]–[53]. A familial study has reported the incidence of mental retardation among the children of first-cousins being four times greater than that in the control group [54]. The study of Morton [51] has revealed that the offspring of first-cousins had over five time’s higher risk of mental retardation when compared to that of the control. The study concluded that decline in IQ or increase in the frequency of mental retardation were consistent with rare recessive alleles associated with around 325 loci, whose likelihood of being transmitted to offspring increases with the relatedness of the parents. The present study differs in several aspects from a previous study conducted among the Ansari Muslims of Bhagalpur [26], as it overcomes the limitations with inclusion of details of inbreeding categories, SES, also five Muslim populations and elaborate statistical models.

Our confirmatory factor analysis, based on ANOVA for the three cognitive domains: VIQ, PIQ and FSIQ, showed significant result in context of inbreeding. Other factors like SES, residence, population, age and gender were found insignificant. Nevertheless, SES and residence have shown significant differences in the mean values of cognitive abilities (Figure 4), though the effects were not sufficient to observe significant effect of inbreeding. This explains the heritability of cognitive abilities was much more influenced by genetic factors (i.e., inbreeding) with least environmental effects. A Dutch twin study has also addressed the changing genetic contribution with age, which displayed the shared environment estimates (c^2^) contributed toward continuity while change in cognition, and the unique environment contributed to change in development [55]. Parental SES and chaos in the home accounted for about 10% or even less of the total variance in the test scores, indicating that they mediate some of the c^2^ effect, but most of that variance still remained unexplained [56]. One of the most recent study has showed a lesser variability in body mass parameters for demographic factors, which may be due to different populations confined to same geographical and environmental conditions explaining the greater extent of their genetic differences [21]. The heritability of intelligence might not be the same for every level of social backgrounds and may be modified by SES [57]. A recent study has revealed that the variation in the intelligence levels among children may be attributed to a greater extent, to individual genetic differences, with almost diminished environmental differences [58].

**Figure 7:**
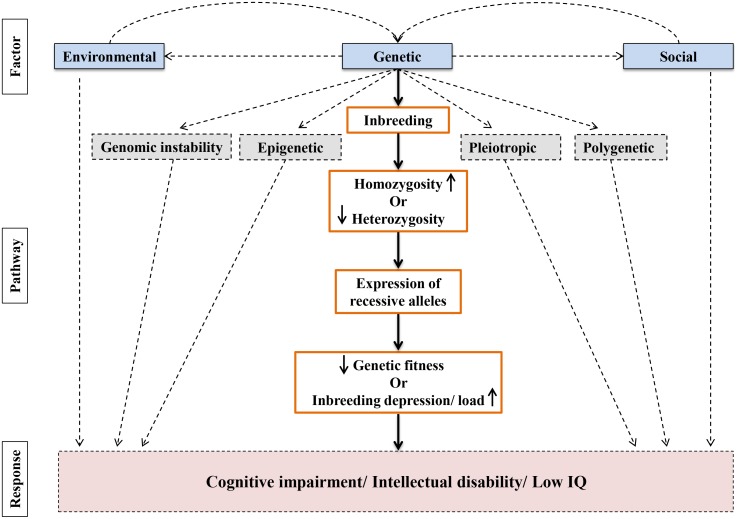
Pathways specifying factors involved in cognitive impairment. The flowchart briefly explains the factors (i.e., environmental, social and genetic) causing cognitive impairment. These factors are inter-related directly or through indirect mechanisms. There are many sub-pathways of each factor, not mentioned here. Inbreeding (due to consanguineous marriages) is also overwhelmed by geography, environment and social norms. Inbreeding increases the homozygosity which in turn results into the expression of autosomal recessive genes that may be one of the causes for abnormal brain development and function.

Figure 7 presents an overview depicting the pathways specifying the factors involved in cognitive impairment with special emphasis on inbreeding. Our simpler model presents the major sources of variance underlying the complex cognitive behavior. It is noteworthy that we have assumed inbreeding, as well as the environmental and social factors for the same cohort to make them rather fit to predict the intelligence more effectively. The phenomenon of inbreeding, undoubtly, is one of the most potent factors to contribute to cognitive behaviors, and also the underlying genetic mechanism. Though heritability of cognitive differences is becoming increasingly clear, the exact nature of the genetic polymorphisms implied by this heritability is still unclear [59]. A recent study based on single nucleotide polymorphism (SNP) predicted that elimination of minor allele leads to no deleterious effect on cognitive ability for a few generations of inbreeding [60].

The recent most techniques provide comprehensive tool to explore the nature and genetics of recessive cognitive disorders [61]. The first homozygosity mapping among Iranian consanguineous families with identification of 8 novel gene loci has revealed heterogeneity of non-syndromic autosomal recessive ID (NS-ARID) [62]. To identify the causative mutation, hundreds of genes were required to be screened among these families, and the gap of knowledge has recently been filled with systematic Sanger sequencing method that led to identification of numerous novel genes for NS-ARID [63]. The next generation sequencing (NGS) has revolutionized the genetic dissection of ID with the identification of gene defects related to ARID and other cognitive disorders. The use of whole-exome enrichment sequencing (WES), a high-throughput NGS technique, has been able to identify for the first time a homozygous variant of *TECR* gene on chromosome 19p13 in a large consanguineous family with NS-ID [64]. In populations where parental consanguinity is common, not all the recessive conditions persist due to autozygous changes, there might be other defects like compound heterozygosity or mutations in intronic, promoter, or other non-coding sequences that could not be detected by WES approach. To unravel the molecular basis of cognitive disorders, candidate gene hunting approach provides substantial novel genes to identify their mechanistic processes. The recently attempted deep sequencing methods reported quite novel candidate genes which are therefore, known to be very attractive candidates for study of synapse- or brain-specific functions, while others involve the basic cellular processes which have been well studied in ID analysis such as DNA transcription and translation, protein degradation, mRNA splicing, energy metabolism and fatty-acid synthesis or turnover [65].

Our study reveals that the extent to which consanguinity affects IQ is proportional to the degree of inbreeding. On the basis of the cognitive study (VIQ, PIQ and FSIQ) outcomes, the offspring of double first-cousins exhibited, on an average, significantly higher inbreeding load (on test scores) than those of first-cousins, which in turn, keep on decreasing with the lowering of inbreeding coefficients and the least of depression being observed among offspring of non-consanguineous parents. Such increasing depression with respect to inbreeding were also reported in previous studies [36], [51], [55], [66]–[68].

### Strengths and Limitations

Our study has immense strengths, though with a few limitations. Firstly, our cohort presents less variation in cognitive abilities for demographic factors, depicting different populations confined to the same environmental conditions. This leaves substantial room for larger study covering different national or geographical regions. A recent study based on national IQs has presented potential significance for demographic, epidemiological and climatic factors [2].

Secondly, the selection of subjects, methodology and statistical analysis can be attempted in a proper way to find out the significant results. ANOVA based factor assessment followed by post-hoc tests provides enough information and the regression analysis provides best measure of cognitive depression in proportion to the degree of inbreeding. However, other statistical tools like oblique rotation method and goodness of fit models can be used as cognitive predictors [69].

Thirdly, standard pedigree information has been used to determine the inbreeding coefficient (F), the extent of inbreeding is related to the amount of ancestry that is shared by the parents of an inbred individual. The pedigree analysis approach adds strength to this study with several advantages: (a) provides natural mating patterns, (b) give accurate estimates of individual inbreeding and its effects and (c) having easy comparisons among studies [41]. However, considerations of more serious methods to study the genetic nature of the recessive cognitive defects such as NGS, WES and candidate gene approaches could provide rather more detailed conclusive proof [61].

## Conclusions

In summary, our comprehensive assessment revealed that parental consanguinity and degree of inbreeding was significantly associated with depression in intellectual behaviors among children. Factors other than inbreeding showed little influence, suggesting that genetic component (i.e., inbreeding) was more influential over these parameters under study. Moreover, the depression in cognitive abilities seems to be more prominent due to increase in the degree of inbreeding (F). This study provides new leads for health care providers and health policy makers to train and make people familiar with the assessment of harmful effects of inbreeding on mental health and cognitive efficiency.

Further studies in context of inbreeding, however, need to be investigated to establish a possible causal relationship between cognitive behavior and so-called generalist genes.
