# Correction: Estimating the Inbreeding Depression on Cognitive Behavior: A Population Based Study of Child Cohort

**DOI:** 10.1371/journal.pone.0115393

**Published:** 2014-12-08

**Authors:** The PLOS ONE Staff

There is an error in the second sentence of the Results section in the Abstract. The sentence should read: The mean differences (95% C.I.) were reported for the VIQ, being −22.00 (−24.82, −19.17), PIQ −26.92 (−29.96, −23.87) and FSIQ −24.47 (−27.35, −21.59) for inbred as compared to non-inbred children (p<0.001).

There is a typo in the fourth sentence of the third paragraph of the Introduction. The correct sentence is: The two key genetic concepts: pleiotropy (in which one gene affects many traits) and polygenicity (in which many genes affect a trait) suggest that there is genetic input into the brain structure and function which is general, not modular [15].
